# Body Size and Weight of Pill Bugs (*Armadillidium vulgare*) Vary between Urban Green Space Habitats

**DOI:** 10.3390/ani13050857

**Published:** 2023-02-26

**Authors:** Shuang Wang, Zhangyan Zhu, Li Yang, Hongshan Li, Baoming Ge

**Affiliations:** 1 Jiangsu Key Laboratory for Bioresources of Saline Soils, School of Wetlands, Yancheng Teachers University, Yancheng 224007, China; 2 College of Biotechnology and Pharmaceutical Engineering, Nanjing Tech University, Nanjing 211816, China; 3 College of Marine and Bioengineering, Yancheng Institute of Technology, Yancheng 224051, China

**Keywords:** soil macrofauna, land cover, urban green space, soil environment

## Abstract

**Simple Summary:**

Urban green areas are critically important for maintaining biodiversity in urban ecosystems. The pill bug is an ecological bio-indicator of soil health that is widely distributed around the world. In this study, we studied the relationship between the characteristics of the pill bugs population and soil properties of urban green spaces. The characteristics of the population vary significantly among selected habitats. The pH and soil organic matter may be the main factors for explaining the variation in body size of pill bugs among the habitats in spring. Significant positive regressions were detected between body length and body weight.

**Abstract:**

Rapid urban development poses a threat to global biodiversity. At the same time, urban green spaces offer opportunities for holding biodiversity in cities. Among biological communities, the soil fauna plays a crucial role in ecological processes but is often ignored. Understanding the effects of environmental factors on soil fauna is critical for ecological conservation in urban areas. In this study, five typical green space habitats were selected including bamboo grove, forest, garden, grassland, and wasteland in spring, for detecting the relationship between habitats and *Armadillidium vulgare* population characteristics in Yancheng, China. Results indicate that soil water content, pH, soil organic matter, and soil total carbon varied significantly among habitats, as well as the body length and body weight of pill bugs. The higher proportion of larger pill bugs was found in the wasteland and the lower proportion in the grassland and the bamboo grove. The body length of pill bugs was positively related to pH. Soil total carbon, soil organic matter, and the number of plant species were correlated with the body weight of pill bugs.

## 1. Introduction

Urban environmental degradation threatens the sustainable development of the human society worldwide, and the degradation occurs not only in developed regions with a high level of urbanization, but also in developing regions with an accelerated urbanization process [1,2,3]. Nowadays, the human society is undergoing the most rapid urbanization in history; many native habitats have been changed to be suitable for human living and have resulted in the homogenization of urban biomes [4,5]. As an important component of urban ecosystems, urban green spaces make an important contribution to biodiversity in urban areas. Meanwhile, urban green spaces also play a vital role in creating recreation for residents and visitors.

Soil fauna is an important ecological indicator for assessing urban soil quality and is involved in many soil functions [6,7,8]. Macrofauna presents an extremely important function in ecosystems, participating in soil carbon transformation, sequestration, nutrient turnover, and maintenance of soil structure, and can be used to evaluate soil and ecosystem quality as well as to assess urban ecosystems [9,10]. With the variations in soil physical, chemical, and microbial properties, the ecological environment of soil macrofauna has changed [11]. Recently, a few studies have proved that the macrofaunal population characteristics are closely related to soil environments, the population characteristics vary among different habitats, and that population conditions can be used as indicators for soil environmental assessment [12,13,14,15].

*Armadillidium vulgare* (Isopoda: Armadillidae), also known as the pill bug, is distributed worldwide, with particularly dense populations in temperate climates, and is one of the most important soil macrofauna in urban green spaces [16,17]. The pill bug population thrives in moist climates and damp soils [18,19,20]. Pill bugs are considered to be detritivores, eating decaying dead leaves and the remains of small invertebrates, and having important functions in soil fertility maintenance, plant nutrient supply, and soil purification, while also feeding on the fine roots and young leaves of plants [16,18,21,22]. They recycle nutrients back into the ecosystem with important ecological significance [16,17,22,23].

Different plant coverage may affect soil environments, such as soil moisture, soil nutrient status, pH, and so on [24,25]. Therefore, we speculate that the population characteristics of pill bugs may relate to soil properties and vegetation types. For instance, soil organic matter is an important food source for pill bugs which may directly affect the distribution of pill bugs [26], which is influenced by land use, land cover, and anthropogenic random factors [27]. However, we did not fully understand the relationship between the pill bugs’ body parameters and soil properties. In this study, we investigated and analyzed the population characteristics of pill bugs among different green spaces on a university campus in Yancheng City, Jiangsu Province. This study aims to address the following two questions: (1) What is the variation pattern of soil properties and population characteristics of pill bugs among different habitats? (2) Which environmental factors can be used to explain the variation in pill bugs’ population?

## 2. Materials and Methods

### 2.1. Study Area

**Figure 1:**
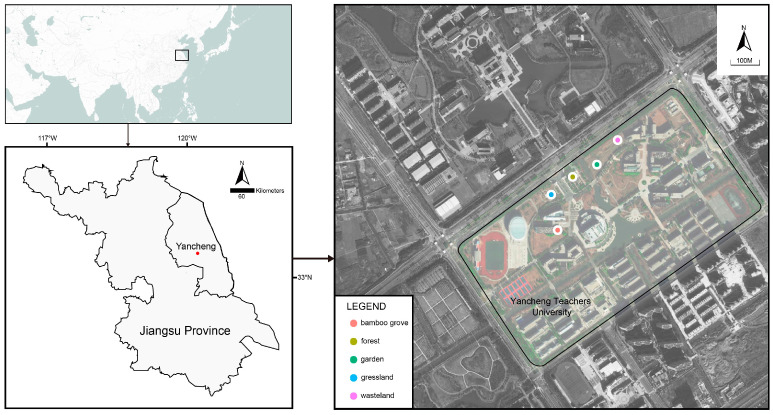
Study area and sampling site of Yancheng City, Jiangsu Province.

We conducted this study on Xinchang campus of Yancheng Teachers University (33.372~33.383° N, 120.193~120.378° E), Jiangsu Province, China (Figure 1). The climate type is region subtropical monsoon, and an average annual temperature of 13.7 °C and an average annual rainfall of 1051 mm was recorded. The campus area is 58.8 ha with a human population of about 12,000. The sampling work was conducted at the beginning of May 2022 in different habitats and finished in a week.

### 2.2. Habitat Selection and Soil Sample Collection

On the campus, we selected five different habitats of green spaces with different land covers, including bamboo grove, forest, garden, grassland, and wasteland. The bamboo grove was dominated by pale bamboo (*Phyllostachys nigra*) with lush growth and partial leaf cover on the ground surface. The forest was mainly dominated by cherry (*Prunus subg. Cerasus sp.*), privet (*Ligustrum lucidum*), and golden raintree (*Koelreuteria paniculata)*. Chinese violet cress (*Orychophragmus violaceus*) was the main vegetation in the garden. The grassland was mainly covered by mascarene grass (*Zoysia tenuifolia)*, bermudagrass (*Cynodon dactylon*), and cogongrass (*Imperata cylindrica*). The wasteland has been unmanaged for about 8 years and has more plant species, including Chinese tallow tree (*Sapium sebiferum*), paper mulberry (*Broussonetia papyrifera*), sweet woodruff (*Galium odoratum*), etc. Five replicates of separate patches were used as sampling sites in each habitat, with a minimum distance of 2 m among the patches in the same habitat. All sampling sites were distributed in an area with a radius of 250 m. The compact distribution of the sampling sites was designed to reduce spatial variability. Details of the habitats are shown in Table 1.

Representative green spaces were selected and randomly sampled, and 5 replicate sample plots (50 cm × 50 cm) were set in each habitat. In each plot, debris such as surface plant and animal residues were removed, and three soil cores (100 cm^3^, depth 5 cm) were taken by using a soil loop cutter. The soil cores were mixed and immediately packed in polyethylene plastic bags. The vegetation coverage and the number of plant species at each sampling site were recorded. The height of the highest plant was measured as the habitat description in each habitat. A total of 25 soil samples were collected from five habitats.

### 2.3. Determination of Soil Properties

Part of the fresh soil samples was used for the determination of soil water content (WC) after oven-drying the samples at 105 °C for 24 h. Part of the fresh soil samples was sieved through an 8-mm sieve and air-dried at room temperature (about 20 °C) for three weeks. The air-dried soils were then sieved through a 2-mm sieve to remove coarse debris and stones. Afterward, the soil samples were used for the determination of electrical conductivity (EC), pH, available phosphorous (AP), and available nitrogen (AN). Then each of the prepared soil samples was ground and passed a 0.149-mm nylon sieve for determination of soil organic matter (SOM), total carbon (TC), and total nitrogen (TN) using the method introduced in the literature [28,29,30,31]. In this research, the used facilities were listed as: a spectrophotometer (LTD. G-9, Nanjing Filer Instrument Co., Nanjing, China) for SOM, AP, and AN, a pH meter (FE 28, Mettler-Toledo Inc. Columbus, OH, USA) for pH, a conductivity meter (Mettler-Toledo FE28, Switzerland) for EC, and a Carbon Nitrogen Elemental Analyzer (Vario Macro Cube, Elementar Analysensysteme GmbH Inc., Langenselbold, Germany) for TC and TN.

### 2.4. Population Survey of Pill Bugs

Pill bugs were captured by searching under fallen leaves, rocks, and topsoil in the selected habitats. They were then placed in bottles in the field and labeled, and were killed and kept by freezing at −20 ℃ in a refrigerator. Then, every individual was thawed and flattened for measurements of body length (*l*) on 1 mm × 1 mm grid paper, and the wet weight (*m*) by an electronic balance.

### 2.5. Statistical Analysis

One-way analysis of variance (ANOVA) was conducted to analyze the soil properties and population characteristics of pill bugs in various habitats. Multiple comparisons were performed using Tukey’s test or Dunnett’s T3 test depending on whether they passed Levene’s test for homogeneity [32]. Principal components analysis (PCA) was performed on soil properties. The applicability of the data to PCA was tested by using the Kaiser-Meyer-Olkin (KMO) sampling adequacy (≥0.500) and Bartlett’s test of sphericity (≤0.050) [30,33,34]. The grouping composition of body length and body weight among different habitats was analyzed by using a Chi-square test, and the post-hoc testing was employed [35,36]. The relationships between environmental factors and physical measurements of pill bugs were analyzed by using Cramer’s V. Mantel test analysis [37]. The relationship between body length and body weight of pill bugs was derived by using regression analysis.

In this study, no variables were transformed. Significant statistical differences were used as *p* < 0.05. A map of sampling sites was created using ArcGIS 10.5 and statistical analyses were performed using Microsoft Office Excel 2021, SPSS18.0, R version 4.2.1, and PAST 4.03 software [38,39,40].

## 3. Results

### 3.1. Soil Properties and Vegetation

Soil properties varied considerably among habitats (Table 2). The WC was highest in the forest at 20.54% and lowest in the grassland at 10.39% (F_4,20_ = 5.908, *p* < 0.01). The forest had the highest pH while the bamboo grove and the garden had the lowest (F_4,20_ = 8.317, *p* < 0.001). There were similar results for SOM (F_4,20_ = 9.587, *p* < 0.001), and TC (F_4,20_ = 8.427, *p* < 0.001), which had their highest values in the bamboo grove and the lowest values in the wasteland. There were significant differences in the number of plant species among habitats, with higher values in the forest and wasteland (F_4,20_ = 16.460, *p* < 0.001). The coverage in the bamboo grove, garden, and grassland was higher, while the coverage in the forest was the lowest (F_4,20_ = 95.667, *p* < 0.001). There were no significant differences in soil TN (F_4,20_ = 1.222, *p* > 0.05), AN (F_4,20_ = 1.458, *p* > 0.05), EC (F_4,20_ = 2.082, *p* > 0.05) and AP (F_4,20_ = 0.594, *p* > 0.05).

**Figure 2:**
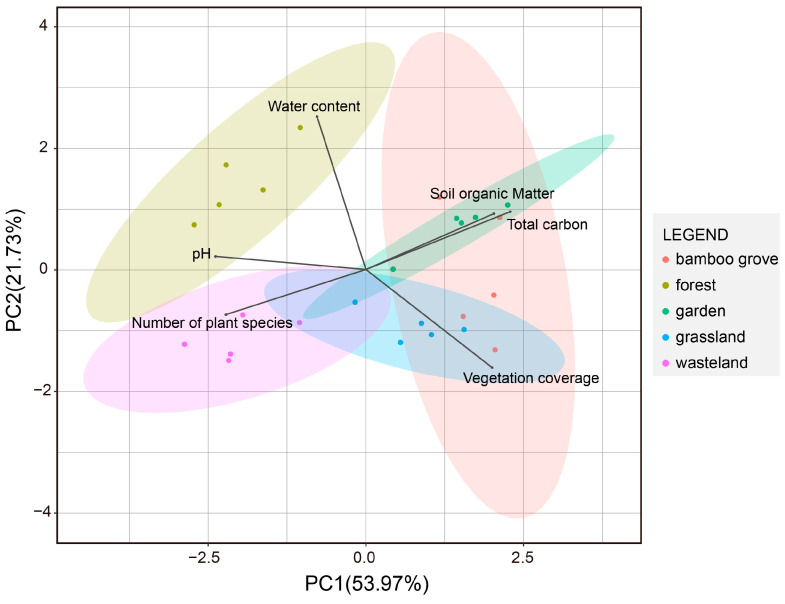
The PCA biplot on soil properties and vegetation.

The overall KMO value of 0.719 (>0.500) demonstrated the adequacy of sampling in this study. The sphericity of Bartlett’s test for PCA showed that these approximately normal data for multiple variables were acceptable (χ^2^ = 62.736, df = 15, *p* < 0.001). Results of PCA showed that soil samples could be classified by habitat characteristics, and pc1 and pc2 could respond to 53.97% and 21.73% of habitat differences (Figure 2). SOM, TC, and vegetation coverage were positively correlated with pc1, whereas WC, pH, and the number of plant species were negatively correlated. Then, pH, WC, SOM, and TC were positively correlated with pc2. However, the number of plant species and vegetation coverage were negatively correlated. The PCA showed that pH had the smallest angle with pc1 and WC had the smallest angle with pc2 The sample plots are clustered to some degree by the habitats in the PCA biplot. The forest is significantly apart from other habitats, and little overlap occurred between the bamboo grove and wasteland and forest. There was no significant separation between the garden, bamboo grove, and grassland.

### 3.2. Characteristics of Pill Bugs’ Population

**Figure 3:**
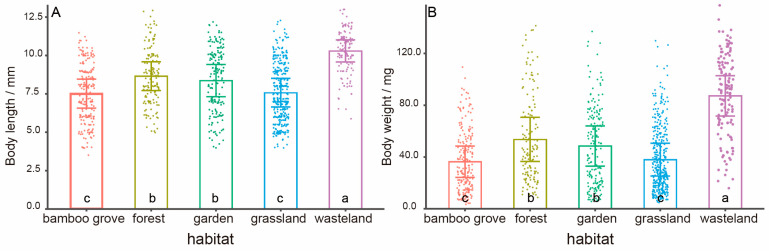
Length (**A**) and weight (**B**) of pill bugs (mean ± S.D.) among different habitats. Note: different letters mean significant differences.

In total, 1015 individuals of pill bugs were captured and measured in our study. The number of individuals was 366 in the grassland, 201 in the bamboo grove, 171 in the garden, and 145 and 132 in the forest and wasteland, respectively. There were significant differences in body length (F_4,1010_ = 61.439, *p* < 0.001) and body weight (F_4,1010_ = 84.835, *p* < 0.001) of pill bugs among habitats. The mean body length and weight of all individuals in the five habitats were (8.21 ± 2.07) mm and (48.06 ± 32.68) mg, respectively. The mean body length and weight in the bamboo grove and grassland were smaller (7.52 ± 1.89) mm (36.34 ± 24.15) mg, (7.58 ± 1.86) mm (37.96 ± 25.33) mg, while the average body length and body weight of wasteland were larger (10.30 ± 1.44) mm (87.27 ± 31.17) mg. The body length and body weight of pill bugs in the forest and the garden were on the medium level (Figure 3A,B).

**Figure 4:**
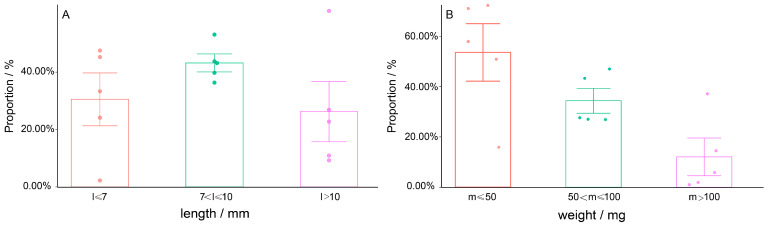
The averaged proportion of length (**A**) and weight (**B**) (mean ± S.D.) in total based on each habitat.

As the specific data obtained from the statistics indicate that most pill bugs were of medium body length. As shown in Figure 4, in total, body length ≤7 mm accounted for 35.5%, 7–10 mm accounted for 43.3%, and *l* > 10 mm accounted for 21.3%. The lighter individuals were the majority. Individuals with body weight ≤ 50 mg accounted for 58.8%, 50~100 mg accounted for 32.4%, and *m* >100 mg accounted for 8.8%. The Chi-square test indicated that the distribution of body length (χ^2^ = 209.325, df = 8, *p* < 0.001) and weight (χ^2^ = 241.131, df = 8, *p* < 0.001) were significantly different among different habitats. There was a moderate correlation between habitat type and body length (Cramer’s V = 0.321, *p* < 0.001), and between habitat type and body weight (Cramer’s V = 0.345, *p* < 0.001).

The post-hoc testing of the grouping composition indicated a higher proportion of bigger individuals (*l* >10 and *m* >100) were found in the wasteland with the highest residuals (>12). Furthermore, the lower proportion of bigger individuals (*l* >10 and *m* >100) were found in the grassland and bamboo grove with the lower residuals (<−4). The variation of the proportion and the residuals are shown in Table 3.

**Figure 5:**
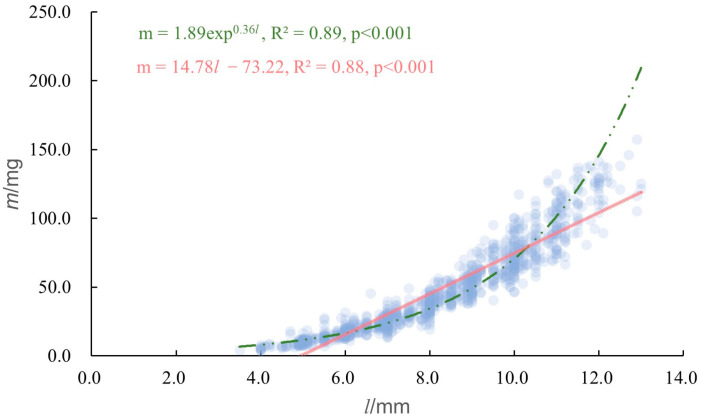
Regressions between body length and body weight of pill bugs.

There was a significant positive regression relationship between body weight (*m*) and body length (*l*) of the pill bugs (Figure 5). The exponential function equation is obtained as *m* = 1.89exp^0.36*l*^ (R^2^ = 0.89, *p* < 0.001) and the linear regression equation is obtained as *m* = 14.78 *l* − 73.22 (R^2^ = 0.88, *p* < 0.001). We found that the power function equation is better able to present the relationship between body weight and body length. There was a significant positive regression relationship between body length and body weight in the selected urban spaces (Figure 5).

### 3.3. Correlation of Environmental Factors with Population Characteristics

**Figure 6:**
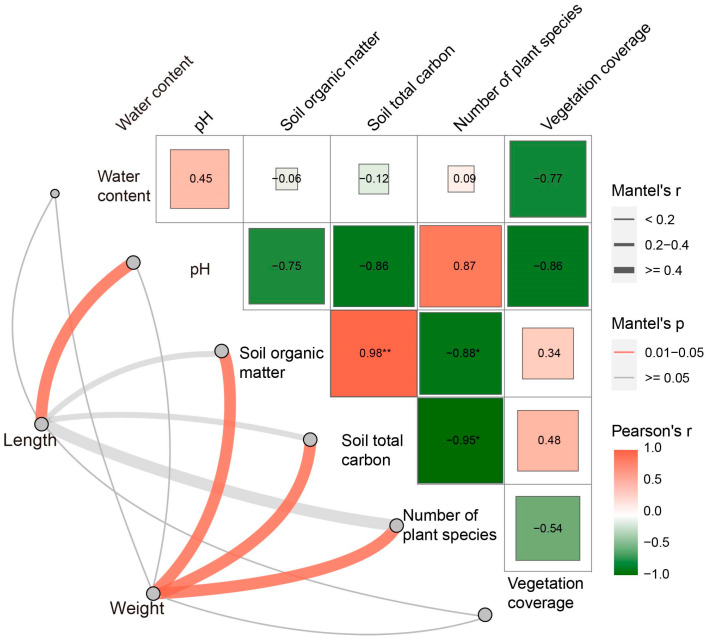
Correlation of habitat environmental factors with physical characteristics of pill bugs (Edge widths indicate Mantel’s r statistics corresponding to distance correlations, and edge colors indicate statistical significance based on 9999 permutations showing pairwise comparisons of physical characteristics with environmental factors. The color gradient in the box indicates Pearson’s correlation coefficient, the magnitude of which is proportional to the r-value, and the number indicates Pearson’s r-value. The “*” means *p* < 0.050; The “**” means *p* < 0.01 for Pearson correlation.)

The body length and body weight of pill bugs was correlated with environmental factors (Figure 6). Results show that pH was strongly related to body length; TC, SOM, and the number of plant species were strongly related to body weight. There was no significant correlation with other environmental factors. The number of plant species presented a significant negative correlation to TC, SOM, and vegetation coverage. A significant positive correlation between SOM and TC was detected in this study. 

## 4. Discussion

### 4.1. Differentiation of Soil Properties among Different Habitats

The impact of rapid urbanization on urban ecosystems has received widespread attention [41]. Changes in urban land use patterns provide opportunities to modify the high heterogeneity of urban green spaces, by scattering different habitat vegetation across urban areas [13]. This helps to mitigate the negative effects of urbanization on native ecosystem biomes, and different vegetation cover also impacts soil physicochemical properties [42,43]. The distribution of some groups of macrofauna has been suggested as a bioindicator of soil quality in previous studies [44,45].

Our study indicates that different vegetation species lead to significant differences in some soil properties, such as SOM and TC. However, soil may keep a high consistency on a relatively small scale, because the soil in the habitats was the same when the campus was constructed in 2005. Although some human activities such as irrigation and fertilization might affect soil properties, no significant differences in the properties of TN and EC among the selected habitats occurred in our study [46,47]. These different manifestations of soil properties may be related to the fact that there are also higher similarities in land use management practices among green spaces [48]. In the artificial environment, the characteristics of the environment tend to be suitable for human habitation and aesthetic needs. Thus, although there are some differences in vegetation in different habitats, the soil environment has not been particularly distinct, showing relatively similar characteristics [49].

### 4.2. Relationship between Environmental Factors and Population Characteristics

There are environmental differences in soil physicochemical properties in urban green spaces [3,50], and pill bugs are more sensitive to environmental changes [26]. Population characteristics can reflect the environmental condition of urban green spaces to a certain extent; hence, the organism can be used as an indicator for the condition of the urban green space ecosystem [51,52]. Pill bugs play an important role in recycling nutrients back into the ecosystem, and present important ecological significance. Furthermore, food quality had important effects on the dynamics of the experimental pill bugs’ populations [53].

Populations of pill bugs exhibited different patterns of life history which may be related to their food resources in previous studies [54]. Studies have shown that there is a certain distribution pattern of soil macrofauna in different habitats and that the soil macrofauna with larger body sizes in habitats received a weaker influence of anthropogenic activities than those with smaller body sizes [55]. In this study, food resources and human disturbances may work together to affect the characteristics of populations. The average body length and weight of pill bugs in the grassland were smaller at (7.58 ± 1.86) mm and (37.96 ± 25.33) mg, respectively, while the average body length and weight of wasteland pill bugs were larger at (10.30 ± 1.44) mm and (87.27 ± 31.17) mg. Characteristics of the grassland and the wasteland were more distinct than those of other habitats (Figure 3). However, we did not collect evidence about the reproduction characteristics of the population, and the species’ reproduction and life cycle may also affect the body size and weight structure of the population [56]. Furthermore, rainfall and temperature also have been mentioned as the influencing factors in the reproduction of pill bugs [54].

In this study, there was a moderately strong correlation between habitat type and pill bugs’ body characteristics (*p* < 0.001), with the larger and heavier individuals in the wasteland *(l* > 10 mm, *m* > 100 mg adjusted labeling residuals all > 12) (Table 3). Meanwhile, soil pH was strongly correlated with body length. TC, SOM, and vegetation species were strongly correlated with body weight (Figure 6). Besides being a key component of good soil structure, increasing the soil capacity of water and nutrient retention, SOM is the primary source of food for the soil biota; it is the base of the soil trophic pyramid. Additionally, the life cycle may present characteristics among habitats because of the variation of the food condition and soil environment [56]. The significant differences in body size and weight among different habitats indicated that the population distribution would be responsive to the soil environment. Especially, soil pH and SOM may be the most important soil properties indicating the distribution of pill bugs. The larger pill bugs tended to be found in habitats with higher soil pH and lower SOM or TC. Though it is a pity that we cannot detect the variations in the life cycle which is related to body size and weight in a single season. However, this should be our next research on variations of the pill bugs’ population with habitat change.

## 5. Conclusions

Changes in soil properties along the vegetation types have a significant effect on the population characteristics of pill bugs. Land use management influenced soil physicochemical properties in urban spaces, and population characteristics changed along with the variation of soil properties. There were significant differences in WC, pH, TC, and SOM of soils among selected habitats, and no significant differences in other soil properties. In addition, the regression equation between body length and body weight was set and well-fitted with a significant positive correlation. The larger pill bugs were found in the wasteland, which may relate to the lower SOM and TC and less disturbance; the smaller pill bugs were found in the grassland and the bamboo grove, which may relate to the lower pH. Furthermore, the food resource and human disturbance may also contribute to the variation of body size and weight among habitats.

## Figures and Tables

**Table 1:** Descriptions of vegetation types in the selected study area.

Vegetation Types	Plant Height (m)	Main Plant Species
Bamboo grove	6.0	*Phyllostachys glauca*
Forest	5.0	*Prunus subg. Cerasus, Ligustrum lucidum, Koelreuteria paniculata*
Garden	0.5	*Orychophragmus violaceus*
Grassland	0.3	*Zoysia tenu ifolia, Cynodon dactylon, Imperata cylindrica, Lolium perenne*
Wasteland	3.0	*Sapium sebiferum, Broussonetia papyrifera, Galium odoratum, Vicia sativa, Sonchus asper*

**Table 2:** Soil properties and vegetation in different habitats (mean± S.D.). Note: different letters in each row mean significant differences while no mark means no significant difference.

Habitat	Bamboo Grove	Forest	Garden	Grassland	Wasteland	Total
WC (%)	12.11 ± 7.36 ^ab^	20.54 ± 3.43 ^a^	18.06 ± 1.37 ^a^	10.39 ± 1.75 ^b^	13.73 ± 2.13 ^ab^	14.97 ± 5.24
pH	6.93 ± 0.05 ^b^	7.17 ± 0.11 ^a^	6.91 ± 0.06 ^b^	6.96 ± 0.15 ^b^	7.12 ± 0.05 ^a^	7.02 ± 0.14
SOM (g kg^−1^)	27.72 ± 1.89 ^a^	22.04 ± 3.75 ^a^	26.40 ± 4.04 ^a^	24.90 ± 1.65 ^a^	13.80 ± 6.59 ^b^	22.96 ± 6.24
TC (g kg^−1^)	25.88 ± 4.76 ^a^	18.90 ± 3.21 ^ab^	25.50 ± 6.10 ^a^	22.62 ± 1.36 ^a^	13.66 ± 2.21 ^b^	21.31 ± 5.89
TN (g kg^−1^)	3.28 ± 0.36	2.72 ± 0.23	3.50 ± 0.60	3.10 ± 0.14	2.94 ± 1.09	3.10 ± 0.61
EC (us cm^−1^)	17.13 ± 1.52	14.61 ± 2.57	16.02 ± 1.23	14.88 ± 3.31	13.53 ± 1.21	15.23 ± 2.32
AN (mg kg^−1^)	11.54 ± 1.40	11.93 ± 1.36	13.48 ± 2.08	10.58 ± 2.15	11.69 ± 2.45	11.84 ± 2.01
AP (mg kg^−1^)	22.38 ± 3.10	24.18 ± 2.63	26.69 ± 2.50	22.95 ± 1.14	22.95 ± 2.55	23.83 ± 2.75
Number of plant species(n)	1.40 ± 0.55 ^b^	5.00 ± 0.71 ^a^	1.60 ± 0.89 ^b^	3.80 ± 0.84 ^ab^	6.00 ± 0.20 ^a^	3.56 ± 2.12
Vegetation coverage(%)	98.00 ± 4.47 ^a^	56.00 ± 5.47 ^b^	96.00 ± 2.24 ^a^	97.00 ± 4.47 ^a^	87.00 ± 2.73 ^a^	86.80 ± 16.63

**Table 3:** The proportion and the residuals (in parentheses) of grouping composition by body length and body weight among different habitats.

	Habitat	Bamboo Grove	Forest	Garden	Grassland	Wasteland	Total
Length/mm	*l* ≤ 7	9.0% (3.2)	3.4% (−3.1)	5.6% (−0.6)	17.1% (6.0)	0.3% (−8.5)	35.5%
	7< *l* ≤ 10	8.7% (0.2)	7.6% (2.6)	6.7% (−1.0)	15.6% (0.0)	4.7% (−1.7)	43.3%
	*l* > 10	2.2% (−4.0)	3.3% (0.5)	4.5% (2.0)	3.3% (−7.0)	8.0% (12.1)	21.3%
Weight/mg	*m* ≤ 50	14.3% (4.3)	8.3% (−0.2)	8.6% (−2.3)	25.6% (5.9)	2.1% (−10.7)	58.8%
	50 < *m* ≤ 100	5.3% (−1.9)	3.9% (−1.3)	7.3% (3.3)	9.8% (−2.7)	6.1% (3.8)	32.4%
	*m* > 100	0.2% (−4.4)	2.1% (2.6)	1.0% (−1.5)	0.7% (−5.8)	4.8% (12.3)	8.8%

## Data Availability

The data presented in this study are available on request from the corresponding author.
